# The Prognostic Value of Neutrophil-to-Lymphocyte Ratio in Metastatic Testicular Cancer

**DOI:** 10.3390/curroncol28010014

**Published:** 2020-12-21

**Authors:** Domen Ribnikar, Igor Stukalin, Philippe L. Bedard, Robert J. Hamilton, Michael Jewett, Padraig Warde, Peter Chung, Lynn Anson-Cartwright, Arnoud J. Templeton, Eitan Amir, Aaron R. Hansen, Daniel Y. C. Heng, Jeremy Lewin

**Affiliations:** 1 Division of Medical Oncology and Hematology, Princess Margaret Cancer Centre, Toronto, ON M5G 2C1, Canada; dribnikar@onko-i.si (D.R.); philippe.bedard@uhn.ca (P.L.B.); Eitan.Amir@uhn.ca (E.A.); Aaron.Hansen@uhn.ca (A.R.H.); 2 Department of Medicine, University of Calgary, Calgary, AB T2N 1N4, Canada; igor.stukalin@gmail.com (I.S.); daniel.heng@ahs.ca (D.Y.C.H.); 3 Princess Margaret Cancer Centre, Department of Surgery, University Health Network, 610 University Ave 3-130, Toronto, ON M5G 2C1, Canada; Rob.Hamilton@uhn.ca (R.J.H.); michael.jewett@uhnresearch.ca (M.J.); 4 Department of Radiation Oncology, Princess Margaret Cancer Centre, Toronto, ON M5G 2C1, Canada; Padraig.Warde@rmp.uhn.ca (P.W.); Peter.Chung@rmp.uhn.ca (P.C.); Lynn.Anson-Cartwright@uhnresearch.ca (L.A.-C.); 5 Department of Oncology, St. Claraspital Basel, and Faculty of Medicine, University of Basel, CH-4058 Basel, Switzerland; arnoud.templeton@kssg.ch

**Keywords:** neutrophil-to-lymphocyte ratio, germ cell tumors, testicular cancer, prognosis, metastatic disease

## Abstract

We investigated the prognostic utility of pre-chemotherapy neutrophil-to-lymphocyte ratio (NLR) in patients with metastatic germ cell tumors (GCTs) undergoing first-line chemotherapy. We utilized two institutional databases to analyze the pretreatment-derived NLR (dNLR). Predictive accuracy was evaluated using the Cox proportional hazard model adjusted for the international germ cell cancer collaborative group (IGCCCG) risk classification. Discriminatory accuracy was evaluated by determining the area under the receiver operating characteristic curve (AUROC). In total, 569 of 690 patients had available dNLR (IGCCCG: good, 64%; intermediate, 21%; poor, 16%). The 5-year and 10-year overall survivals (OSs) for good, intermediate, and poor risk groups were 96.2%, 92.8%, and 62.7% and 93.9%, 90.3%, and 62.7%, respectively. A dNLR of 2 provided the best discriminatory accuracy with an AUROC of 0.58 (95% CI: 0.52–0.65, *p* = 0.01) for progression-free survival (PFS), whereas for OS, a dNLR of 3 provided the best discriminatory accuracy with an AUROC of 0.62 (95% CI: 0.53–0.70, *p* < 0.01). A dNLR > 2 was associated with a hazard ratio (HR) of 1.99 (95% CI: 1.27–3.12, *p* < 0.01) for PFS, which lost its effect after adjustment for IGCCCG (HR: 1.44, 95% CI: 0.90–2.30, *p* = 0.13). For OS, a dNLR >3 was associated with an HR of 3.00 (95% CI: 1.79–5.01, *p* < 0.01), but lost its effect after adjustment for IGCCCG. Systemic inflammation plays a role in metastatic GCT, but its prognostic utility beyond established algorithms is limited. The general prognostic value of NLR can be seen across a number of tumors, although the consistency and magnitude of the effect differ according to cancer type, disease stage, and treatment received. We identified that an elevated NLR was associated with an adverse PFS and OS, but not independent of the IGCCCG risk classification. dNLRs >2 and >3 were associated with an adverse PFS and OS, respectively, in patients with metastatic GCT receiving first-line chemotherapy, but not independent of the IGCCCG risk classification.

## 1. Introduction

Since the development of the international germ cell cancer collaborative group (IGCCCG) classification, patients with metastatic germ cell tumors (GCTs) are traditionally treated with three or four cycles of bleomycin, etoposide, and cisplatin (BEP) in good or intermediate/poor risk disease, respectively [1]. This risk-stratified approach utilizing clinical parameters (site of primary tumor, location of metastatic lesions, histological subgroup, and degree of tumor marker elevation) was initially reported to have 5-year survival rates of 91%, 79%, and 48% for good, intermediate, and poor risk groupings, respectively, and is the classification scheme commonly used in patient selection for GCT clinical trials. However, it has been increasingly apparent that even though the original classification included data from over 5000 patients, refinement of IGCCCG may be required for which work has recently been presented [2,3]. Recent studies demonstrate improvement in survival, particularly in the IGCCCG intermediate and poor risk groups [4,5,6,7]. The basis for the improvement is thought to be related to the centralization of patient care in high-volume centers of excellence, in addition to advances seen in salvage treatments, supportive care, and aggressive surgical management of residual masses [8]. Thus, the real prognostic significance of the IGCCCG classification is being challenged, and attempts to create improved risk stratification are underway [2,3,9,10].

The interplay between the tumor microenvironment and the host inflammatory response influences the development and progression of cancer [11]. Easily measurable blood-based parameters that reflect systemic inflammation include elevation of C-reactive protein, leucocytes, cytokines, and platelets and reduction in protein and albumin [12]. Evolving data have identified that an elevated ratio of peripheral neutrophils to lymphocytes (NLR), a marker of host inflammation, is a consistent poor prognostic factor across multiple cancer types and disease stages [11]. However, the influence of NLR in patients with metastatic GCT is underinvestigated, with preliminary reports suggesting that systemic inflammatory markers may be of prognostic significance [13,14].

Although cisplatin-based chemotherapy has revolutionized the treatment for metastatic GCT, approximately 10%–15% of patients relapse after first-line therapy. Identifying novel approaches that add value to the established prognostic IGCCCG classification is important to optimize care for GCT patients. Thus, the aim of this study was to independently evaluate the significance of pre-chemotherapy NLR as a prognostic factor for patients with metastatic GCT beyond the IGCCCG classification.

## 2. Methods

### 2.1. Participants and Measures

We included patients with metastatic GCT who received first-line chemotherapy between 1 January 1990 and 31 December 2013 at two institutions (Princess Margaret Cancer Centre (PM), Toronto, ON, Canada; Tom Baker Cancer Centre (TBCC), Calgary, AB, Canada). Institutional research ethics board approval was granted prior to data collection at both sites, and patients were identified from GCT databases. The baseline characteristics collected included IGCCCG risk group, chemotherapy regimen, pre-chemotherapy tumor markers, treatment details, and relapse characteristics, including progression-free survival (PFS) and overall survival (OS). Patients with mature teratoma on post-chemotherapy lymph node dissection were not considered to have relapsed. Absolute white blood count (WBC; 10^9^/l) and neutrophil count (10^9^/l) before chemotherapy were used to calculate the derived NLR (dNLR; calculated as the absolute neutrophil count divided by the total white blood cell count minus the absolute neutrophil count; previously shown to have a prognostic value similar to NLR) [15]. Only patients with complete dNLR data were included in the outcome analysis. PFS was measured from the date of initial chemotherapy until disease progression or death from any cause. OS was measured from the date of initial chemotherapy until death from any cause. Salvage treatments, including the use of high-dose chemotherapy, were up to the clinical decision making of the individual institution. The patients were censored at the date of the last follow-up.

### 2.2. Statistical Analysis

Data were reported descriptively as proportions, medians, and ranges as appropriate. The predictive accuracy of dNLR was evaluated using the Cox proportional hazard analysis and reported as hazard ratios (HRs) together with their respective 95% confidence intervals (CIs). Analyses were then adjusted for the IGCCCG risk classification. The discriminatory accuracy was evaluated by determining the area under the receiver operating characteristic curve (AUROC) for survival at 5 years. The optimal cut-off for NLR selection was chosen based on the highest seen AUROC. All statistical analyses were conducted using SPSS statistical software, version 21 (IBM Corp., Armonk, NY, USA). All statistical tests were two-sided, and statistical significance was defined as *p* < 0.05. No corrections were made for multiple statistical testing.

## 3. Results

### 3.1. Patient Characteristics

A total of 690 patients with metastatic GCT treated with first-line chemotherapy were identified (475 at PM and 215 at TBCC). The patients’ demographic characteristics are listed in Table 1. There were no major differences in baseline demographic features between the two cohorts. The median age was 31 (range, 16–85). Overall, proportions of the primary tumor site and IGCCCG risk groups were concordant with the literature, with 64%, 21%, and 16% of the patients classified as good, intermediate, and poor risk, respectively. All the patients received platinum-based chemotherapy with the most common regimen being BEP × 3 given in 239 (35%) patients, followed by BEP × 4 in 249 (36%), EP × 4 in 136 (20%), and VIP × 4 in 17 (2%) patients.

### 3.2. Outcome

**Figure 1:**
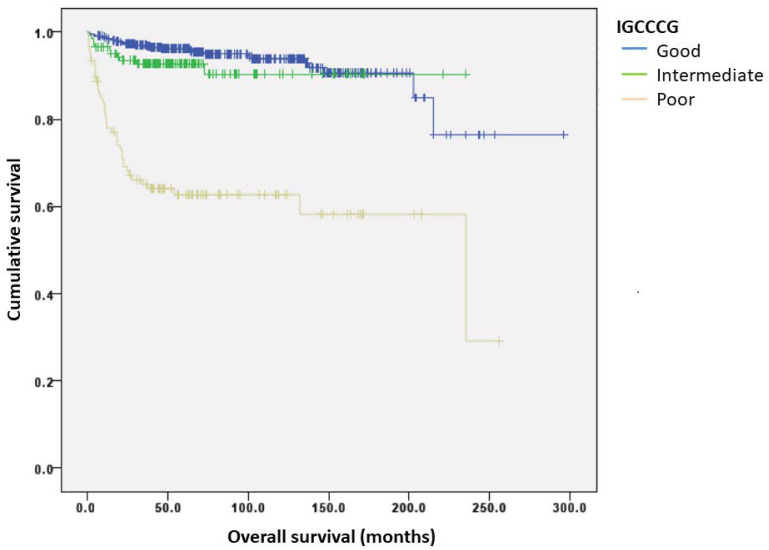
Kaplan–Meier curves of overall survival (OS) according to the IGCCCG group.

**Figure 2:**
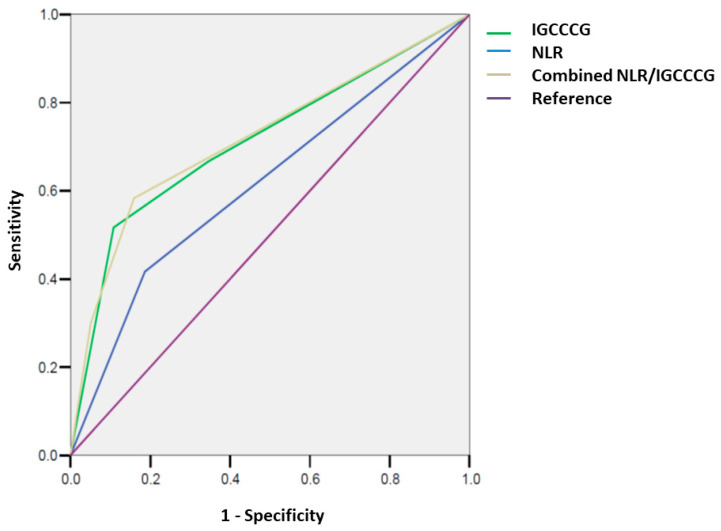
Discriminatory accuracy (AUROC) of the IGCCCG group and NLR, separate and combined for overall survival (OS).

A total of 107 (16%) patients had progressed and 77 (11%) patients died at a median follow-up of 64 months. Outcome assessment was conducted on those with complete data available for dNLR analysis (*n* = 569). The 5-year PFSs for good, intermediate, and poor risk groups were 91.8%, 85.4%, and 59.2%, respectively, while the 10-year PFSs were 89.2%, 85.4%, and 56.8%, respectively. The 5-year OSs for good, intermediate, and poor risk groups was 96.2%, 92.8%, and 62.7%, respectively, while the 10-year OSs were 93.9%, 90.3%, and 62.7%, respectively (Figure 1). The discriminatory accuracies of IGCCCG were 0.68 (95% CI: 0.61–0.75) for PFS and 0.72 (95% CI: 0.64–0.80) for OS (Figure 2).

### 3.3. dNLR

Next, we investigated the prognostic utility of dNLR for PFS and OS as a continuous variable (*n* = 569). The HRs for PFS and OS were 1.09 (95% CI: 1.03–1.16, *p* < 0.01) and 1.14 (95% CI: 1.07–1.21, *p* < 0.01), respectively. For PFS, a dNLR cut-off of 2 provided the best discriminatory accuracy with an AUROC of 0.58 (95% CI: 0.52–0.65, *p* = 0.01) (Supplementary Materials, Table S1), whereas for OS, a dNLR cut-off of 3 provided the best discriminatory accuracy with an AUROC of 0.62 (95% CI: 0.53–0.70, *p* < 0.01) (Supplementary Materials, Table S2).

On univariable analysis, a dNLR > 2 was associated with a hazard ratio (HR) of 1.99 (95% CI: 1.27–3.12, *p* < 0.01) for PFS. On multivariable analysis, this effect was lost when adjusted for IGCCCG risk (HR: 1.44, 95% CI: 0.90–2.30, *p* = 0.13), while the discriminative accuracy using the established IGCCCG was maintained particularly in patients with poor risk disease (HR: 4.87, 95% CI: 2.93–8.08, *p* < 0.01).

When investigating OS outcomes, on univariable analysis, a dNLR > 3 was associated with an HR of 3.00 (95% CI: 1.79–5.01, *p* < 0.01) (Table 2), but this effect was lost after adjustment for IGCCCG risk group (HR: 1.59, 95% CI: 0.91–2.77, *p* = 0.10). However, IGCCCG risk maintained its discriminative significance particularly in high-risk disease with an HR of 6.73 (95% CI: 3.67–12.35, *p* < 0.01), potentially confirming dNLR as a surrogate marker of tumor burden (Table 2).

Among patients with IGCCCG high risk, the median OS was 47.6 months if the dNLR ≤ 3, while if the dNLR > 3, the median OS was reduced to 39.6 months. In a univariable analysis for OS within the IGCCCG high risk, a dNLR > 3 was associated with an HR of 1.46 (95% CI: 0.71–2.98, *p* = 0.31). 

### 3.4. Correlations

Spearman’s ρ for the correlation between IGCCCG risk group and NLR as a continuous variable was 0.306 (*p* < 0.01).

## 4. Discussion

Highly effective cisplatin-based chemotherapy has dramatically improved outcomes for patients with metastatic GCT. Treatment is based on the IGCCCG risk classification, which was developed based on clinical outcome data from over 5000 patients prior to the year 1990. However, it has been increasingly recognized that further refinement of the model may be required given the significant advances in diagnostic approaches, improved salvage treatment, aggressive surgical management of residual masses, and improved supportive care. Similarly, the initial model captured patients treated in an era prior to the widespread application of BEP as initial chemotherapy and may be less prognostic in certain clinical scenarios (e.g., IGCCCG intermediate risk group based on LDH alone). Highlighting the general improvement of outcomes for GCT, an updated meta-analysis in patients with non-seminoma reported 5-year OSs of 94%, 83%, and 71% in good, intermediate, and poor risk disease, respectively, compared with 91%, 71%, and 48% within the original cohort risk groups. This is reinforced by outcomes presented in the IGCCCG Update Consortium for both non-seminoma (96%, 88%, and 67% for good, intermediate, and poor risk disease, respectively) and seminoma (95% and 87% for good and intermediate risk disease, respectively) [1,2,3,4]. Our study, which has contributed data to the IGCCCG Update Consortium and is based on metastatic GCT patients treated between 1990 and 2014, demonstrated 5-year OSs of 96%, 93%, and 63%, respectively, highlighting the need to identify better prognostication and treatment particularly in the poor risk group.

Our data suggest that systemic inflammation plays a role in metastatic GCT, but its prognostic utility beyond established algorithms is limited. We identified that an elevated NLR was associated with an adverse PFS and OS, but not independent of the IGCCCG risk classification. It is worth noting that NLR is one of the many potential inflammatory prognostic biomarkers, which include C-reactive protein, hypoalbuminemia, elevated LDH, and platelet-to-lymphocyte ratio. The general prognostic value of NLR can be seen across a number of tumors, although the consistency and magnitude of the effect differ according to cancer type, disease stage, and treatment received [16,17,18,19]. A meta-analysis of 100 studies involving more than 40,000 patients investigated the prognostic impact of NLR and identified a median cut-off of elevated NLR of 4, which was associated with an adverse overall survival (HR = 1.81) [11]. However, the optimal cut-off for the utilization of NLR remains ill-defined with variable definitions in the literature [11]. In our study, we assessed NLR as a continuous variable and identified that cut-offs of 2 and 3 provided the best discriminatory accuracy for PFS and OS, respectively, lower than the median cut-off of 4 reported in the literature [11].

The distinct mechanisms in the association between high NLR and worse outcomes are not entirely understood, although the link between inflammation and cancer progression is well established [20]. A pro-inflammatory state driven by high neutrophil counts is associated with suppression of lymphocytes and activated T cells, which may lead to reduced targeted apoptotic cancer cell death via impaired cytotoxic T cells [21,22]. In addition, higher cytokine release (in particular IL-1 and IL-6) leads to enhanced tumor development and increased pro-metastatic behavior [23]. In particular, serum IL-6 has been identified as elevated across a number of tumor types and is associated with a negative prognostic effect and higher tumor stage [24].

To date, limited studies have investigated the value of NLR on outcomes in GCT. Most studies have assessed its role in the pre-orchiectomy setting, where a higher NLR may be associated with advanced cancer staging and worse overall outcomes [25,26,27]. To our knowledge, a single study by Fankhauser et al. assessed the prognostic utility of systemic inflammatory markers, including NLR, in 146 patients receiving first-line chemotherapy for GCT [14]. Interestingly, on multivariable analysis, they identified that leukocyte count, neutrophil count, NLR, and systemic immune-inflammation index were all independent prognostic factors beyond the IGCCCG risk groups when comparing good/intermediate vs. poor risk. Differences between the findings of our study and the report by Fankhauser may be related to our larger sample size or the differences within the patient populations being described, although the proportion of patients with poor risk GCT was 16% for both cohorts [14]. The allure of utilizing NLR is that it can be derived prior to the initiation of chemotherapy; thus it is a readily available approach for assisting prognostication. However, contrary to Fankhauser et al., we did not demonstrate independent prognostic utility beyond IGCCCG and hypothesize that it may simply be a reflection of tumor burden [14,28].

We acknowledge a number of limitations of our study. First, the study is retrospective in design and will need further confirmation in studies where blood work has been collected prospectively or within the updated IGCCCG risk classification. Second, we included blood work within variable time frames (within 21 days) from the date of initial blood draw to the start of chemotherapy. Third, neutrophil and lymphocyte counts are nonspecific parameters, which may be influenced by other concomitant medical conditions, such as infection, venous thromboembolism, and other systemic inflammatory processes. Although the patients included in the cohort were heterogeneous and not controlled for treatments received in the salvage setting, both institutions have standardized approaches in the management of GCT, including the role of post-chemotherapy resection of residual masses and availability of high-dose chemotherapy.

## 5. Conclusions

In summary, an NLR greater than 2 and 3 was associated with an adverse PFS and OS in patients with metastatic GCT receiving first-line chemotherapy; however, this was not independent of the IGCCCG risk classification. At this stage, further investigation is required to evaluate the role of NLR as a cost-effective prognostic biomarker in metastatic testicular cancer.

## Figures and Tables

**Table 1:** Baseline patients’ clinical characteristics.

	PM (*n* = 475)	TBCC (*n* = 215)	Total (*n* = 690)
Characteristic (Number, %)			
Median age, years (interquartile range)	31 (16–85)	31 (16–61)	31 (16–85)
Chemotherapy regimen *			
3 × BEP	141 (30)	98 (46)	239 (35)
4 × BEP	189 (40)	60 (28)	249 (36)
4 × EP	102 (21)	34 (16)	136 (20)
4 × VIP	10 (2)	7 (3)	17 (2)
Other	31 (7)	16 (7)	47 (7)
Primary tumor site			
Testis	459 (97)	215 (100)	674 (98)
Retroperitoneal	2 (1)	0	2 (1)
Mediastinum	11(2)	0	11(2)
Other	3 (1)	0	3 (1)
IGCCCG risk group			
Good	308 (65)	131 (61)	439 (64)
Intermediate	94 (20)	50 (23)	144 (21)
Poor	73 (15)	34 (16)	107 (16)

* BEP: bleomycin, etoposide, cisplatin; EP: etoposide, cisplatin; VIP: ifosfamide, etoposide, cisplatin. PM = Princess Margaret Cancer Centre; TBCC = Tom Baker Cancer Centre.

**Table 2:** Univariable and multivariable analyses for overall survival (OS) according to dNLR > 3.

	Univariable			Multivariable		
Variable	HR	95% CI	*p*-Value	HR	95% CI	*p*-Value
NLR > 3	3.00	1.79–5.01	<0.01	1.59	0.91–2.77	0.10
IGCCCG risk						
Low	Ref			Ref		
Intermediate	1.52	0.75–3.08	0.25	1.34	0.61–2.96	0.47
High	8.09	4.93–13.28	<0.01	6.73	3.67–12.35	<0.01

## Supplementary Materials

The following is available online at https://www.mdpi.com/1718-7729/28/1/14/s1: Table S1. Determination of optimal cut-off of NLR for PFS according to discriminatory accuracy evaluated by determining the area under the receiver operating characteristic curve (AUROC). Table S2. Determination of optimal cut-off of NLR for OS according to discriminatory accuracy evaluated by determining the area under the receiver operating characteristic curve (AUROC).
