# Supplementary material for: The Prognostic Value of Neutrophil-to-Lymphocyte Ratio in Metastatic Testicular Cancer
Source: Curr Oncol. 2020 Dec 21;28(1):107–14. doi: 10.3390/curroncol28010014 (PMC7816171; doi:10.3390/curroncol28010014)
Supplement: Supplementary file 1 [file curroncol-28-00014-s001.zip › curroncol-1017408supp.pdf]

Supplementary material

Table S1. Determination of optimal cut-off of NLR for PFS according to discriminatory accuracy evaluated by determining the area under the receiver operating characteristic curve (AUROC). .

NLR cut-off	AUROC	95% CI	P value
**2**	0.58	0.52 - 0.65	0.01
**3**	0.57	0.50 - 0.64	0.04
**4**	0.55	0.48 - 0.62	0.12
**5**	0.53	0.46 - 0.60	0.42
**6**	0.52	0.45 - 0.59	0.62
**7**	0.51	0.44 - 0.58	0.74

Table S2. Determination of optimal cut-off of NLR for OS according to discriminatory accuracy evaluated by determining the area under the receiver operating characteristic curve (AUROC). .

NLR cut-off	AUROC	95% CI	P value
2	0.58	0.50 - 0.66	0.05
3	0.62	0.53 - 0.70	<0.01
4	0.57	0.49 - 0.66	0.06
5	0.55	0.47 - 0.63	0.21
6	0.54	0.46 - 0.62	0.30
7	0.53	0.45 - 0.61	0.45
